# The Value of Exercise Electrocardiography in Outpatients with Stable Chest Pain and Low Pre-Test Probability of Significant Coronary Artery Disease

**DOI:** 10.3390/jcm12144670

**Published:** 2023-07-14

**Authors:** Pontus Thorild, Georgios Mourtzinis

**Affiliations:** 1 Department of Medicine and Emergency Mölndal, Sahlgrenska University Hospital, 431 80 Mölndal, Sweden; pontus.thorild@vgregion.se; 2 Institution of Medicine, Sahlgrenska Academy, University of Gothenburg, 405 30 Gothenburg, Sweden

**Keywords:** stable chest pain, angina pectoris, coronary artery disease, exercise ECG, pre-test probability

## Abstract

The role of exercise electrocardiography (ECG) in the investigation of stable chest pain has been questioned. The American Heart Association guidelines suggest the use of exercise ECG in patients with stable chest pain and low pre-test probability (PTP) of significant coronary artery disease, while the European Society of Cardiology Guidelines does not. This retrospective observational study aimed to assess the usefulness of exercise ECG in the low-PTP population with stable chest pain. We reviewed the medical records for all outpatient exercise ECGs conducted because of stable chest pain at the Department of Medicine and Emergency, Sahlgrenska University Hospital, Mölndal, Sweden, during 2016–2018. The identified patients were categorized in low-, intermediate-, or high-risk pre-test probability of significant coronary artery disease. All low-PTP patients were followed for one year post investigation for the incidence of acute coronary syndrome and all-cause mortality. Thus, 505 patients (mean age 60 years, 56% women) with low PTP were included in the study. Only four patients (0.6%) experienced incident myocardial infarction (three patients) or all-cause mortality (one patient). The negative predictive value of exercise ECG was 99.7%, and the positive predictive value was 28.6%. In this low-PTP population, exercise ECG yields a good negative predictive value and a poor positive predictive value.

## 1. Introduction

The valuation of stable chest pain in outpatients remains a diagnostic challenge on a daily basis for many physicians. Although the cause of chest pain is often noncardiac, coronary artery disease needs to be ruled out [1]. One of the biggest challenges is deciding which patient should be investigated with which method. The guidelines from the American Heart Association (AHA) from 2021 and the guidelines from the European Society of Cardiology (ESC) from 2020 suggest the use of the modified Diamond and Forrester model of pre-test probability (PTP) of significant coronary artery disease [1,2,3]. The PTP assessment is based on a patient’s symptoms, sex, and age, and patients are stratified as high risk, intermediate risk, and low risk of significant coronary artery disease. High-risk patients are recommended to undergo a further invasive investigation. Intermediate-risk patients are recommended to undertake a non-invasive diagnostic test (non-invasive functional imaging for myocardial ischemia or non-invasive anatomical imaging). In low-risk patients, the ESC guidelines suggest no further investigation, while the AHA guidelines propose that exercise testing without imaging, i.e., exercise electrocardiography (ECG), is a reasonable test for excluding myocardial ischemia and determining functional capacity. Therefore, there is a disparity between the two guidelines in the low-risk population, derived from scarce knowledge and variety in studied populations. In this study, we aimed to evaluate the usefulness of exercise ECG in outpatients with stable chest pain and low PTP of significant coronary artery disease.

## 2. Materials and Methods

This study is a retrospective observational outcome analysis. We identified all outpatients who had undergone exercise ECG at the Department of Medicine and Emergency, Sahlgrenska University Hospital, Mölndal, Sweden, during 2016–2018. Patients were identified in the hospital’s booking software by procedure code AF012 (exercise ECG, standard). Thereafter, the authors performed a structured review of the hospital’s electronic patients’ medical records for all the identified patients, a database created with all the outpatients that underwent exercise ECG because of stable chest pain. Stable chest pain was defined as not new-onset and without increasing in severity and intensity.

All exercise ECGs were performed at the same unit with the same standardized clinical procedure. In that unit, the exercise ECG is satisfactory when patients reach 85% of the maximum predicted rise in heart rate. Criteria for positive exercise ECG include abnormal ST-segment horizontal or downsloping depression of >1 mm, ST-segment elevation >1 mm in non-Q wave lead, onset of ventricular arrhythmia, development of new bundle branch block, chest pain, and fall in systolic blood pressure (>20 mmHg). After the exercise has stopped, recording continues for up to 4 min.

The date of the exercise ECG was considered the index date. If a patient went through multiple examinations with exercise ECG during this three-year period, then only the first one was included. The collected baseline variables included age, sex, indication for exercise ECG (chest pain/dyspnea), known ischemic heart disease (previous myocardial infarction, percutaneous coronary intervention or coronary artery bypass graft), hypertension, hyperlipidemia (pharmacologically treated), diabetes (pharmacologically treated), congestive heart failure, and smoking habits (ongoing smoking). Exercise ECG results were categorized as negative, inconclusive, or positive, respectively, and recorded without exception, as assessed in the medical record. Included patients were classified according to the modified Diamond and Forrester model of PTP in three groups: high risk, intermediate risk, and low risk of significant coronary artery disease [4]. Classification was based on nature of symptoms (typical, atypical, non-anginal, or dyspnea), sex, and age in accordance with the model presented in the ESC guidelines for the diagnosis and management of chronic coronary syndromes from 2020 [2].

All patients in the low-PTP population were included in the final analyses. Data were collected from the electronic patients’ medical record about further investigations and the results of them, as well as if investigations resulted in revascularization (percutaneous coronary intervention or coronary artery bypass graft). All those patients with low PTP were followed-up in the electronic medical record one year after the index exercise ECG for incident acute coronary syndrome (ACS), i.e., myocardial infarction or hospitalization for unstable angina, and all-cause mortality. The electronic medical record is updated for myocardial infarction and hospitalization for unstable angina if the patient is still living in the same region of Sweden, and updated for all-cause mortality if the patient is still living in Sweden. This study was approved by the Swedish Ethics Review Authority (# 2022-00033-01). Patient consent for participation was not obtained nor deemed necessary.

Data are presented as proportions or as mean values ± standard deviation (SD), as appropriate. Negative predictive value is calculated as the ratio of number of true-negative exercise ECGs to number of negative exercise ECGs [5]. True negative is defined as without myocardial infarction or death during the one year of follow-up. Positive predictive value is calculated as the ratio of number of true-positive exercise ECGs to number of positive exercise ECGs. True positive is defined as significant coronary artery disease treated with revascularization after the index stable chest pain investigation. All analyses were conducted using IBM SPSS statistics Version: 28.0.1.0.

## 3. Results

**Figure 1:**
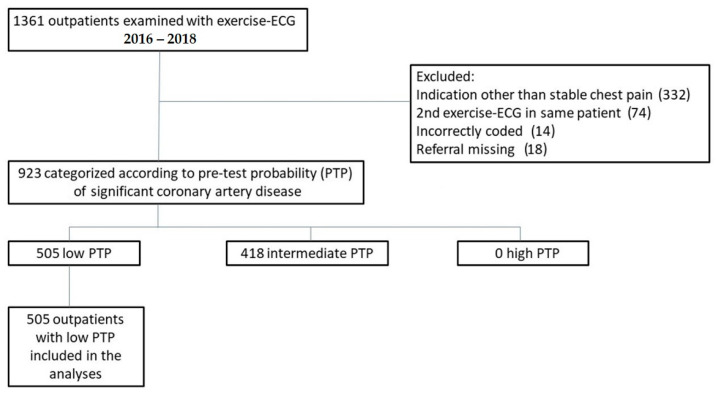
Flowchart showing the derivation of the study population. ECG = electrocardiography. PTP = pre-test probability.

During 2016–2018, a total of 1361 outpatients had registered exercise ECG in the hospital’s booking software, and their electronic medical records were reviewed in this study. Thus, 438 examinations were excluded, as shown in Figure 1, and we identified 923 outpatients who had been examined with exercise ECG because of stable chest pain. Further, 505 patients (55%) were classified as low PTP and 418 patients (45%) classified as intermediate PTP. There were no high-risk patients identified.

### 3.1. Low Pre-Test Probability Patients

**Figure 2:**
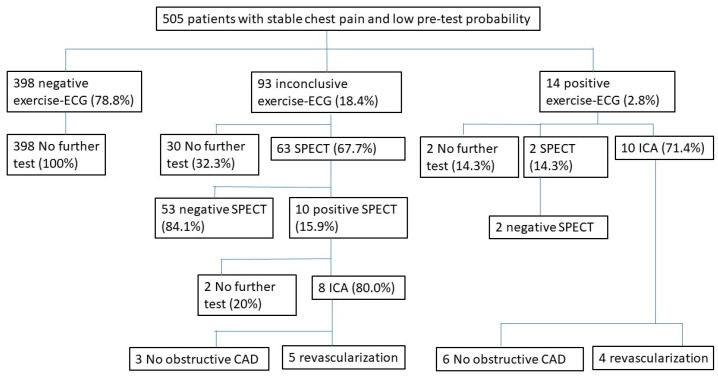
Initial test result, follow-up test, and revascularization. ECG = electrocardiography; SPECT = single-photone emission computed tomography; ICA = invasive coronary angiography; CAD = coronary artery disease.

In this study, 505 outpatients (mean age 60 years, 56% women) with low PTP of significant coronary artery disease were included in the analyses of this study. The baseline characteristics of those 505 outpatients are presented in Table 1. In this low-PTP group, the exercise ECG was negative in 398 patients (78.8%), and those patients did not undergo any other test. Also, 93 patients (18.4%) had inconclusive exercise ECG, and 14 patients (2.8%) had positive exercise ECG; most of them underwent further examination that resulted in revascularization in 5 patients (5.4%) of the inconclusive exercise ECG group and in 4 patients (28.6%) in the positive exercise ECG group (Figure 2).

### 3.2. One-Year Outcome in Low Pre-Test Probability Patients

All 505 patients within the low-PTP group were followed-up one year after the index exercise ECG. Only 4 (0.8%) out of 505 patients met the endpoint of ACS (3 patients) or all-cause mortality (1 patient). The outcomes in relation to exercise ECG results are presented in Table 2. The negative predictive value of exercise ECG was 99.7%, and the positive predictive value was 28.6%. In a sub-analysis of the low-PTP population without known coronary artery disease, only 1 out of 347 patients (0.3%) with negative exercise ECG experienced an endpoint, ACS, during the follow-up (negative predictive value 99.7%). No death occurred in this sub-group during the follow-up. Among the 51 patients with known coronary artery disease at baseline and negative exercise ECG, only 1 experienced an endpoint (negative predictive value 98.0%).

## 4. Discussion

We conducted a retrospective observational study in 505 low-PTP outpatients according to PTP of significant coronary artery disease who underwent exercise ECG because of stable chest pain. The main findings are that, in this population, exercise ECG yields a good negative predictive value (99.7%) and a poor positive predictive value (28.6%) for one-year incident ACS or all-cause mortality. To our knowledge, there are no previous reports on the use of exercise ECG in low-risk patients with stable chest pain stratified by the current PTP model.

Overall, this studied population with low PTP of clinically significant coronary artery disease had low rates of endpoints in the form of ACS or all-cause mortality. This is in line with the position that the contemporary PTP of significant coronary artery disease across symptomatic patient categories is substantially lower than currently assumed [4]. Of the whole studied population, only four patients (0.8%) met the combined endpoint during the follow-up, although 23.2% of the population had known coronary artery disease. The same incidence of ACS or all-cause mortality was found in the sub-populations with and without known coronary artery disease. Most of the studied population (78.8%) had a negative exercise ECG and were not in need of further investigation. Although only 73 patients (14.5%) in this study had known coronary artery disease, a negative exercise ECG in this sub-population seems to have the good predictive value as well. Among the 107 patients without negative exercise ECG, i.e., inconclusive or positive, 9 patients (8.4%) underwent revascularization because of clinically significant coronary artery disease as a result of further chest pain investigation. This is the clinical health gain that can be achieved by investigating this low-PTP population.

Previous studies in unselected populations have shown that exercise ECG is better at excluding than confirming coronary artery disease [6]. In addition, the exercise ECG can give valuable additional clinical information; patients with intermediate risk of significant coronary artery disease who achieved 85% or greater of their maximum age-predicted heart rate and a high exercise capacity have very low rates of cardiac mortality and nonfatal myocardial infarction [7]. In patients with suspected coronary artery disease, positive exercise ECG approximately doubled the risk of adverse endpoints in subjects with normal perfusion and further increased the risk, even if perfusion was abnormal in myocardial perfusion single-photon emission computed tomography [8]. Furthermore, with rising healthcare costs, there is a need to widely distribute limited resources [9]. Appropriate patient selection is very important to prevent patients at low risk of unnecessary interventions or delaying others at high risk. Exercise ECG can, in specific populations, offer a low-cost rule-out test in low-risk patients [10]. This study enlightens the possible value of negative exercise ECG in the low-PTP population, with and without known coronary artery disease.

Similar outcomes after exercise ECG in low-risk patients have been described from the Mayo clinic, although without stratifying the patients according to the PTP [11]. Results from the What Is the Optimal Method for Ischemia Evaluation in Women trial showed a similar low incidence of outcomes after exercise ECG in women with a low risk of coronary artery disease but with lower rates of negative exercise ECG [12]. A post hoc analysis in stable chest pain patients in the SCOT-HEART trial showed that exercise ECG had similar NPV (96%) and PPV (38%) for detecting prognostically significant obstructive coronary artery disease, although without PTP stratifying the population [13]. It is, though, worth mentioning that both the SCOT-HEART trial and other trials, e.g., the PROMISE trial, suggest that computed tomography coronary angiography is more accurate than the exercise ECG to detect or rule out coronary artery disease in patients with stable chest pain [14]. However, the aim of the current study was not to compare chest pain investigation modalities but to assess the usefulness of exercise ECG, as proposed in the AHA guidelines for the Evaluation and Diagnosis of Chest Pain from 2021 [1]. AHA guidelines suggest that exercise ECG is a reasonable investigation in patients with stable chest pain with low PTP and without known coronary artery disease (class of recommendation 2a). In exactly this low-PTP population, our study showed a good NPV of 99.7% and, thus, justifies the use of exercise ECG as a first- or second-line option in this population. Moreover, the current study extends the same results in the low-PTP population with stable chest pain and with known coronary artery disease. In this population with known coronary artery disease, it is not recommended to investigate chest pain with computed tomography coronary angiography due to the high risk of false-positive results [1]. This is of importance since exercise ECG is a widely available low-cost test, while other investigation modalities may not be accessible for all patients.

There are, though, some limitations that we need to point out. First, we were not able to compare exercise ECG with other modalities or no investigation at all. Not to investigate low PTP patients is an approach that is also recommended as an option by the current AHA and ESC guidelines. With that approach, however, a proportion of patients with significant coronary artery disease would be undiagnosed (8.4% in our study). Secondly, the study is based on patients who were referred for exercise ECG. Unfortunately, we do not know how this referral selection was made. This issue may contribute to referral bias. Third, a longer follow-up time might reveal more false-negative results. However, even computed tomography coronary angiography without the presence of obstructive (>50%) coronary artery disease has incrementally increased annualized major cardiovascular event rates [15]; thus, there is a potential need to re-investigate in the case of symptoms after one year. Fourth, the study has an observational retrospective design with the limitation that the physicians did not follow a study protocol. Therefore, some patients with positive tests did not undergo further investigation but received pharmacological therapy (physician decision). Unfortunately, we did not have complete access to patients’ medical therapy. Fifth, the event rate in the studied population was low, as expected.

## 5. Conclusions

In conclusion, we reported the usefulness of exercise ECG in a population with stable chest pain and low pre-test probability of significant coronary artery disease. We found that exercise ECG in this population yields a good negative predictive value and a poor positive predictive value. These results, in line with the ESC guidelines, show that exercise ECG in this population is a reasonable alternative test to rule out significant coronary artery disease. Furthermore, these results extend the AHA recommendations to the population with stable chest pain, low pre-test probability, and known coronary artery disease. 

## Figures and Tables

**Table 1:** Baseline characteristics of 505 outpatients with stable chest pain and low pre-test probability of significant coronary artery disease.

All Patients	N = 505
Mean age, years (±SD)	59.6 (±13.6)
Women	284 (56.2%)
Coronary heart disease	73 (14.5%)
Current smoking	38 (7.5%)
Hypertension	196 (38.8%)
Hyperlipidemia	123 (24.4%)
Diabetes mellitus	50 (9.9%)
Heart failure	7 (1.4%)

Data are numbers unless otherwise indicated. SD = standard deviation.

**Table 2:** One-year outcomes among 505 outpatients with chest pain and low pre-test probability of significant coronary artery disease in relation to exercise ECG result.

	Exercise ECG Results		
	Negative	Inconclusive	Positive
Acute coronary syndrome	2 (0.4%)	0	1 (0.2%)
All-cause mortality	0	0	1 (0.2%)

Data are numbers unless otherwise indicated. ECG = electrocardiography.

## Data Availability

The data underlying this article cannot be shared publicly due to ethical and legal restrictions from Swedish authorities. Data can, however, upon reasonable request to the authors and with permission from the Swedish National Board of Health and Welfare, be available to researchers who meet the criteria for access to confidential data.
